# Nurses’ job preferences on the internet plus nursing service program: a discrete choice experiment

**DOI:** 10.1186/s12912-023-01692-0

**Published:** 2024-02-15

**Authors:** Yan He, Guanrui Feng, Chenchen Wang, Dan Yang, Lei Hu, Wai-kit Ming, Weiju Chen

**Affiliations:** 1 Department of Infectious Diseases and Public Health, Jockey Club College of Veterinary Medicine and Life Sciences, City University of Hong Kong, 5/F, Block 1, To Yuen Building, 31 To Yuen Street, Tat Chee Avenue, Kowloon, Hong Kong China; 2 School of Nursing, Jinan University, No. 601, Huangpu Avenue West, Guangzhou, Guangdong China; 3 Department of Public Health and Preventive Medicine, School of Medicine, Jinan University, Guangzhou, Guangdong Province China; 4 Department of Pharmacy, International school, Jinan University, Guangzhou, China; 5 Department of Endodontics, Stomatological Hospital of Guangzhou Medical University, Guangzhou, China

**Keywords:** Internet plus nursing service program, Home health nursing, Job preference, Discrete choice experiment

## Abstract

**Background:**

The Internet Plus Nursing Service (IPNS) is being instigated in all provincial-level regions throughout China, in which registered nurses (with more than five years of experience from qualified medical institutions) will provide services to those in their communities or homes after receiving online applications. The growing shortage of human resources in nursing is a critical issue for this project, so effective policies for recruiting and retaining nurses are critical.

**Objective:**

This study aims to pinpoint the significant job characteristics that play a crucial role in shaping the job decisions of sharing nurses in the IPNS program, and to estimate the strength of job attributes.

**Methods:**

A discrete choice experiment (DCE) was used to assess job attributes influencing sharing nurses’ preferences. A qualitative design, including in-depth interview and focus interview methods, was conducted to determine the inclusion of attributes. The final included six attributes were: work modes, duration per visit, income per visit, personal safety, medical risk prevention, and refresher training. This study was conducted at 13 hospitals in Guangdong Province, China, from April to June 2022, and a total of 220 registered sharing nurses participated in the survey. The multinomial logit model explored attributes and relative valued utility. Preference heterogeneity is explored via latent class analysis (LCA) models.

**Results:**

A total of 220 participants answered the questionnaire. Income was the most influential characteristic of a sharing nursing position, followed by personal safety management, duration per visit, medical risk prevention, and refresher training, and nurses’ preferences differed among different types of classes.

**Conclusions:**

Sharing nurses place most value on income and personal safety with career-related decisions, which indicates an urgent need to develop complete security for personal safety. This study can be helpful to decision-makers in the Chinese government.

## Introduction

With the rapid growth of the aging population [1], there is an increasing demand for home health care services [2, 3]. By 2030, there will be more than 400 million people over 65 [4], many of whom suffer from multiple health problems, including loss of capacity, disabilities, and noncommunicable chronic diseases [5, 6]. Those with complex health problems need a range of home health care services, such as wound care, health education, injections, and illness monitoring.

Home health care (HHC) is defined as services offered in the home to individuals and their families to improve their quality of life [7, 8]. A wide range of regions and countries around the world have been providing this kind of care. More than 4.9 million patients in the United States receive HHC services, and the population continues to grow [9]. In Europe, between 1 and 5% of the public health budget is spent on these services [10]. In Japan, people can order home visits via the care website, and the rising costs can be covered by health insurance [11, 12].

HHC services are mainly delivered by community nurses in health service institutions [13]. Nevertheless, the lack of geriatric professionals and home care nurses makes it challenging [14]. In response to this situation, the National Health Commission issued a “Notice on promoting the Pilot Program of the ‘Internet Plus Nursing Service (IPNS)’ program” in February 2019, and six provinces were designated as pilot cities [15]. This program facilitates the provision of home health care through internet technology (i.e., mobile apps, online websites, or internet-based platforms) and medical institutions, which is also called “The Sharing Nurse Program” [16]. In 2020, a “Notice on Furthering the Pilot Project of ‘Internet Plus Nursing Service to was officially established for the national spread of the IPNS program [15].

The target population of the IPNS program is community-dwelling older adults, especially those who are seriously ill or have mobility limitations [15]. Clients register on the apps, e.g., WeChat, and order medical services according to their health needs. After assessing the client’s situation, nurses arrive at the client’s home with a medical kit for the ordered services. The providers are certified nurses from professional medical agencies (i.e., hospitals and health centers) and arrange home visits with their daily work. The IPNS program consists of two main core components, daily care, and medical care, with tasks like exchanging tubes, administering medications and injections, and traditional Chinese medical care.

One of the significant challenges of facilitating the IPNS program is the shortage of qualified nurses. Home care differs from nusing services offerd in medical institutions, meaning nurses from hospitals or community health agencies need extensive training to deliver effective home care services. The government should develop economical and non-economical strategies to motivate qualified nurses to join the IPNS program. Previous IPNS-based research [17] focused on the willingness of nurses to join this program and found that Chinese nurses are generally optimistic about this. However, it is unclear what type of incentives nurses value most in an Internet Plus Nursing Service job, and how this preference differs by type of individuals.

To formulate effective recruitment and retention strategies, it is necessary to consider the main job characteristics that influence sharing nurses’ decisions. Therefore, this study aims to (1) utilize qualitative methods to identify job characteristics of the IPNS program that affect sharing nurses’ job decisions, (2) use a discrete choice experiment (DCE) to provide life-like job scenarios and examine the relative strength of job attributes. The findings of this study can provide relevant information for the development of sharing nurses retention strategies, thus helping to address the challenges faced by the IPNS program in terms of staff shortages and high turnover. Moreover, this research explores the heterogeneity of preferences among sharing nurses, which draws attention to the need for personalized policies that are tailored to individual needs.

## Method

We used the DCE methodology to determine factors influencing sharing nurse job preferences with characteristics of the latent classes. DCE is a quantitative technique in health economics for healthcare products and programs [18]. According to relevant literature, we assume respondents’ preferences with job choices largely depended on features that comprise attributes of the experiment [19, 20]. Respondents selected preferences by considering attributes, as well as simulating actual scenarios.

### Development of attributes and levels

Following DCE guidelines [21], a qualitative approach could ensure that the attributes and levels of the questionnaire simulated realistic scenarios. This development was conducted in two phases.

First, we reviewed the literature on career decisions related to home health care and the IPNS program to identify which attributes might be relevant. There is little literature on sharing nurses’ decisions, and Internet-based home care services differ from traditional home care in terms of work mechanisms and many other aspects; therefore, we conducted a qualitative study to fill the gap. Semi-structured interviews were held with 11 nurses by the researcher (YH) for one month. Participants were asked to delineate job factors influencing their decisions and to explain them with examples from home visiting experiences. Attributes and levels of inclusion in the DCE questionnaire were defined through focus interviews. We consulted with experts and nursing consultants about the accuracy and applicability of the initial attribute levels, while some adjustments and modifications were made. Six attributes and corresponding levels were finalized, which is shown in Table 1.

**Table 1:** Attributes and levels

Attributes	Description given to respondents	Levels
Working modes	Refer to types of home care, include part-time working and full-time working. Nurses are affiliated to a qualified medical institution in both models.	L1 Part-time work
		L2 Full-time work
Duration per visit	Refer to duration of a single visit (including round-trip transport)	L1 1 h
		L2 2 h
		L3 3 h
		L4 4 h
Income per visit (¥)	Refers to income earned from a single visit (net revenue after removal of platform overheads and round-trip transport costs)	L1 100
		L2 250
		L3 400
		L4 550
Personal safety management	Refers to security measures to ensure the nurses’ personal safety, including clients information verification, alarm equipment, real time position tracking, recording of the service procedure and other measures.	L1 Superior (Two nurses involved in each visit with advanced security measures offered.)
		L2 Medium (One nurse involved in each visit with sufficient security measures offered.)
		L3 Basic (One nurse involved in each visit with basic security measures offered.)
Medical risk prevention	Refers to the identification, assessment and management of medical risks, such as work structures, workload manageability.	L1 Superior (Sufficient medical risk prevention mechanisms which could identify potential risks and improves them.)
		L2 Medium (Nearly sufficient medical risk prevention mechanisms comply with relevant regulations)
		L3 Basic (Insufficient medical risk prevention mechanisms which do not comply with the relevant regulations.)
Refresher training	Refers to strength of trainings that occur after the initial qualification provided to nurses to support their knowledge and skills-building.	L1 Less than 4 h
		L2 4 − 8 h
		L3 More than 8 h

After determining the attributes and levels, the experimental design of a DCE should be carefully constructed to ensure that it is representative, and efficient. A total of 864 (2 × 4 × 4 × 3 × 3 × 3) scenarios were generated for this study, as it was impractical to have numerous scenarios, requiring a factorial design to identify the most representative [22]. So the Lighthouse Studio module of Sawtooth software was used for an orthogonal experimental design, which ensures good representativeness. We created different combinations of scenarios by the choice-based conjoint (CBC) function, with all versions being horizontally balanced and orthogonal [20]. We also set up an opt-out option to allow participants to be able to opt out of the two choice options offered.

The DCE is a survey-based research method and the research instrument is mainly the DCE questionnaire, which includes a description of the job choice scenario, a demographic informatics survey, and a DCE experiment with introductions to the attributes and levels. This is a web-based questionnaire with original data on the secure server of Sawtooth software (Provo, Utah, USA) [23, 24]. Before starting, 20 sharing nurses used the DCE questionnaire for a pre-survey. After completing the questionnaire independently, they were consulted about whether to consider if any changes were necessary. The final version of the questionnaire is shown in Table 2.

**Table 2:** Schematic diagram of DCE choice question

options	Scenario A	Scenario B	Neither
Working mode	full-time work	Part-time work	choose neither A nor B
Duration per visit	1 h	4 h	
Income per visit (¥)	550	100	
Personal safety management	Superior (Two nurses involved in each visit with advanced security measures offered.)	Basic (One nurse involved in each visit with basic security measures offered.)	
Medical risk prevention	Superior (Sufficient medical risk prevention mechanisms which could identify potential risks and improves them.)	Basic (Insufficient medical risk prevention mechanisms which do not comply with the relevant regulations.)	
Refresher training	More than 8 h	Less than 4 h	
Which scenario would you choose	select	select	select

### Sample and data collection

The target population of this study is sharing nurses who have participated in the IPNS program. Eligibility criteria for nurses to participate included: (a) obtaining the People’s Republic of China Nurse Practitioner Certificate; (b) registering and being certified as a sharing nurse on the IPNS information platform of medical institutions; (c) clinical nursing practice for over five years.

We conducted the study in Guangzhou, one of China’s first six pilot cities for the IPNS program. Invitations were sent to the heads of hospitals and health care facilities that launched this program. After permission, researchers went to the medical facilities to distribute questionnaires. Convenience sampling was used to invite nurses who met the following criteria to participate in the study. Respondents were informed of the purpose of this study and instructions for DCE, to scan the QR code and complete it.

The sample size of the study can be calculated by the following formula [18]: N > 500c/(t × a).

The sample size (N) of the DCE main effect was determined by the selection tasks (t = 12), the alternatives (a = 2), and units in the quantitative analysis (c = 4); we calculated that the sample size required for this study had to be greater than 83.

A total of 13 hospitals (including 11 tertiary and 2 secondary hospitals) accepted our invitation to participate in the research. This study was conducted from April to June 2022 for two months. The process did not identify information from any respondents, yet each answered questions voluntarily.

### Statistical analysis

#### Multinomial logit model

A multinomial logit model was used to analyse the relative factors influencing nurses’ preferences in Internet Plus Services. The multinomial logit model (MNL) was based on random utility theory, as respondents chose alternatives to maximize it, including how people will trade off between attributes. This related participants’ choices to different attributes in every scenario and we statistically analyzed the preference weight captured in the questionnaire. The choices acted as utility indicators, given that the utility is a latent construct [18]. The positive or negative coefficients showed the direction of respondents’ preferences. The utility weight was determined for each component attribute, assuming that systematic utility is the sum of its parts. The following formula estimated the likelihood that a participant would select one from the set:


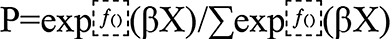


where x might be an attribute and an economic aspect for the participant.

#### Latent class analysis model

To treat heterogeneity in random preferences, we used the latent class analysis (LCA) model, widely implemented in longitudinal studies, to find characteristics of classes by class allocation probabilities dependent on predictors [25]. Both attribute importance and relative valued utility were explored to analyze the trade-offs participants made between attributes. A semiparametric approach modeled the relational structure of data. We identified distinct classes of preference modes in our sample. Based on Akaike Information Criterion 3 (AIC3) and Bayesian Information Criteria (BIC), the optimal classes were determined in an iterative procedure by comparing models and the number of classes [26] for unobserved heterogeneity in individual behavior [27].

#### Willingness to pay (WTP)

This is what an individual will pay for a change in the attribute linked to money, and measures the willingness to receive an increase or decrease in pay for a better option and/or to avoid a worse option [18, 28]. We used a ratio of the coefficient of an attribute to the price attribute in determining willingness to pay for a change under two fictitious situations: this determined how much a person prefers a specific level of the same attribute [29].

## Results

A total of 220 of 231 nurses were enrolled. Four nurses (1.73%) declined to participate in the study and 7 (3.03%) did not finish all questions, which were considered exclusions from the analysis, so the response rate was 95.24%.

The average age of the respondents was 35 years, ranging from 19 to 56 years. Of the 220 participants, 213 (96.82%) were female, 80.00% were married, more than half (64.99%) had a bachelor’s degree and 55.00% obtained a position as a chief nurse or higher. The majority in our sample had relatively extensive work experience (59.55% had worked for 10 years or more), and 40.91% had at least two years of work experience as a sharing nurse. The demographic information of the participants is shown in Table 3.

**Table 3:** Basic demographic informatics of participants

Demographic informatics	N(%)
Gender	
Male	7 (3.28%)
Female	213 (96.82%)
Educational Background	
Secondary school diploma	32 (14.54%)
Junior college degree	45 (20.45%)
Bachelors degree	129 (58.63%)
Master degree	14 (6.36%)
Marital status	
Unmarried	38 (17.27%)
Married	176 (80.00%)
Other	6 (2.73%)
Years of service	
5–9 years	89 (40.45%)
10–14 years	59 (26.82%)
15–19 years	26 (11.82%)
≥20 years	46 (20.91%)
Position titles	
Junior nurse	16 (7.27%)
Senior nurse	83 (37.73%)
Chief nurse	83 (37.73%)
Vice nurse consultant	36 (16.36%)
Nurse consultant	2 (0.91%)
Length of time as an Internet nurse	
Less than 1 years	51 (23.18%)
1–2 years(including 1 years)	79 (35.91%)
2–3 years(including 2 years)	69 (31.36%)
More than 3 years	21 (9.55%)
Monthly income ^a^	
< ¥ 2000	1 (0.45%)
¥ 2000 - ¥ 3999	16 (7.27%)
¥ 4000 - ¥ 5999	54 (24.55%)
¥ 6000 - ¥ 7999	30 (13.64%)
¥ 8000 - ¥ 9999	42 (19.09%)
> ¥ 10,000	77 (35.00%)

^a^ The currency exchange rate of ¥1 = US $0.16 is applicable

### Utility report for the multinomial logit analysis model

The positive and negative coefficients indicated whether people liked the attribute level, as shown in Table 4. Sharing nurses preferred the part-time mode (β = 0.05) than the full-time mode (β=-0.05), and they preferred 1-hour (β = 0.29) and 2-hour (β = 0.14) duration per visit. In addition, participants demonstrated a strong preference for 400 (β = 0.33) and 500 (β = 0.81) income per visit. They showed a similar preference for highest levels of personal safety management (β = 0.56) and medical risk prevention (β = 0.22) compared to the medium level. Refersher training more than 8 h was also important, but to a relatively low degree (β = 0.06).

**Table 4:** Utility of each attribute level and the result of logit analysis (N = 220)

Attributes and levels	Utility	Coefficient	Standard Error	P value	Odds ratio	95% CI
Working mode						
Part-time work	6.99	0.05	0.03	0.08	Reference	
Full-time work	-6.99	-0.05	0.03	0.08	0.90	(0.86–0.96)
Duration per visit						
1 h	41.05	0.29	0.06	< 0.001	Reference	
2 h	19.10	0.14	0.06	0.01	0.85	(0.77–0.95)
3 h	-4.91	-0.04	0.05	0.51	0.72	(0.65–0.80)
4 h	-55.24	-0.40	0.06	< 0.001	0.50	(0.45–0.56)
Income per visit(¥)^a^						
100	-139.00	-0.99	0.06	< 0.001	Reference	
250	-20.12	-0.14	0.05	0.007	2.34	(2.11–2.60)
400	46.53	0.33	0.05	< 0.001	3.77	(3.39–4.19)
550	112.59	0.81	0.06	< 0.001	6.05	(5.41–6.75)
Personal safety management						
Superior	78.56	0.56	0.05	< 0.001	Reference	
Medium	4.88	0.03	0.05	0.44	0.59	(0.54–0.64)
Basic	-83.44	-0.60	0.05	< 0.001	0.31	(0.29–0.35)
Medical risk prevention						
Superior	30.15	0.22	0.04	< 0.001	Reference	
Medium	0.66	0.01	0.05	0.92	0.81	(0.74–0.88)
Basic	-30.81	-0.22	0.04	< 0.001	0.65	(0.59–0.71)
Refresher training						
Less than 4 h	-6.44	-0.05	0.04	0.30	Reference	
4 − 8 h	-2.30	-0.02	0.05	0.72	1.03	(0.94–1.13)
More than 8 h	8.75	0.06	0.04	0.16	1.11	(1.02–1.22)

^a^ The currency exchange rate of ¥1 = US $0.16 is applicable

Overall, We found that the six attributes with the highest utility were part-time working mode (β = 0.05), 1 h of duration per visit (β = 0.29), 550 income per visit (β = 0.81), superior personal safety management (β = 0.56), superior medical risk prevention (β = 0.22), and more than 8 h of refresher training (β = 0.06). The most important of these attributes was 550 income per visit (β = 0.81). Among the attributes negatively correlated with sharing nurses’ job preferences, 100 income per visit (β=-0.99), basic personal safety management (β=-0.60), and basic medical risk prevention (β=-0.22) were the least popular attributes.

P values less than 0.05 were considered statistically significant. All levels in “Income per visit” were statistically significant (for all levels P < 0.05). Also statistically significant were levels other than 3 hours duration per visit (P = 0.51), levels other than medium level of personal safety management (P = 0.44), and levels other than medium medical risk prevention(P = 0.92).

The odds ratio is commonly used in epidemiological studies; we obtained the value by comparing the first level with other levels for the same attribute to determine if people disliked or liked it. When the ratio was less than 1, it indicated that people preferred the reference level, while the opposite was true if the ratio was more significant than 1. Sharing nurses preferred the part-time work mode, while the estimated preference weight for full-time work mode was negative. As the level of income per visit and refresher training attributes increased, sharing nurses were more preferred to them. As working hours increased, personal safety management and medical risk prevention decreased, sharing nurses more resistant.

**Fig. 1:**
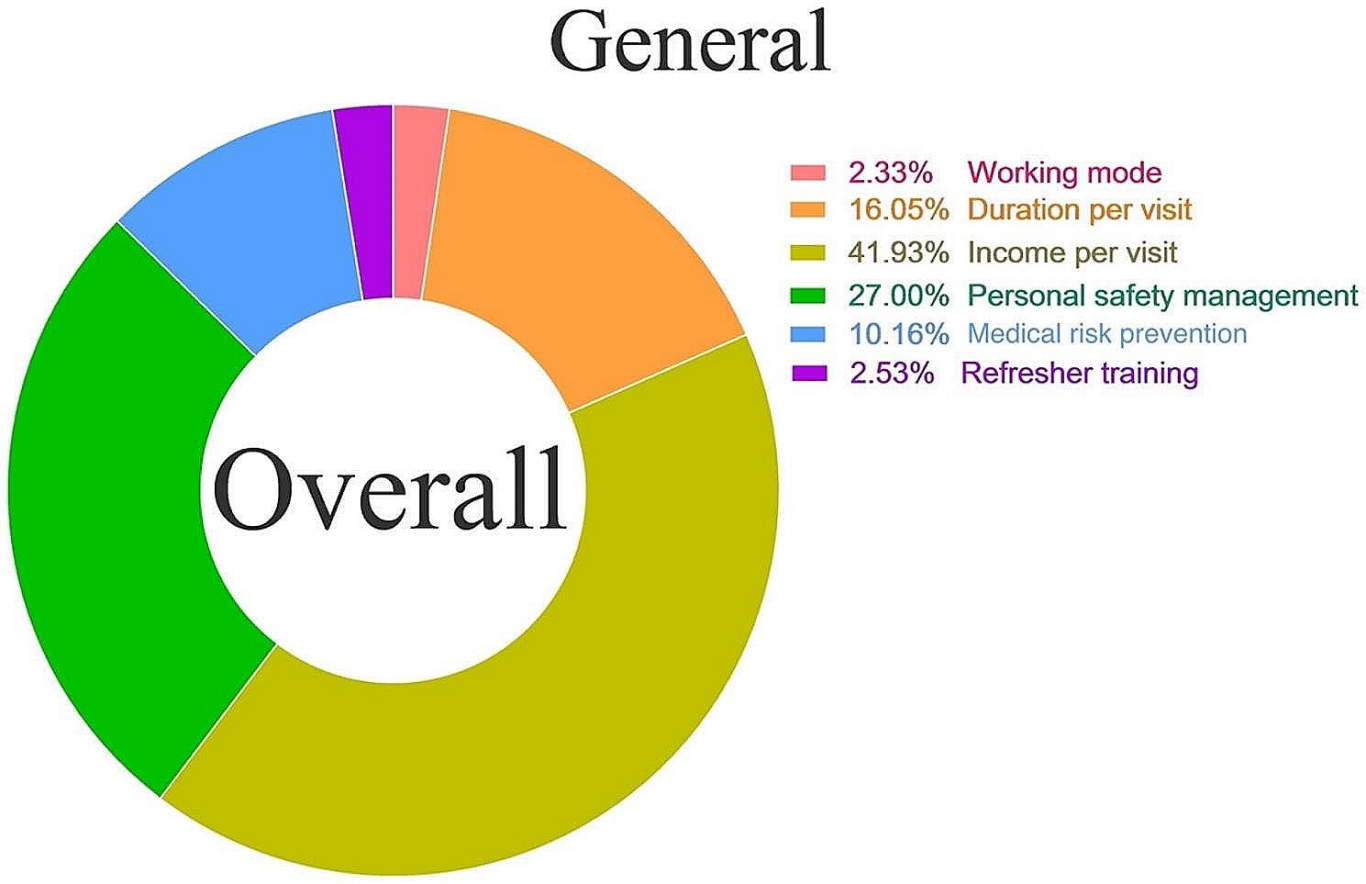
The proportion of each attribute for general three classes

In the preferences of sharing nurses (Fig. 1), the most important was income per visit (41.93%), followed by personal safety management (27%), and duration per visit(16.05%).

### Latent class analysis model

**Fig. 2:**
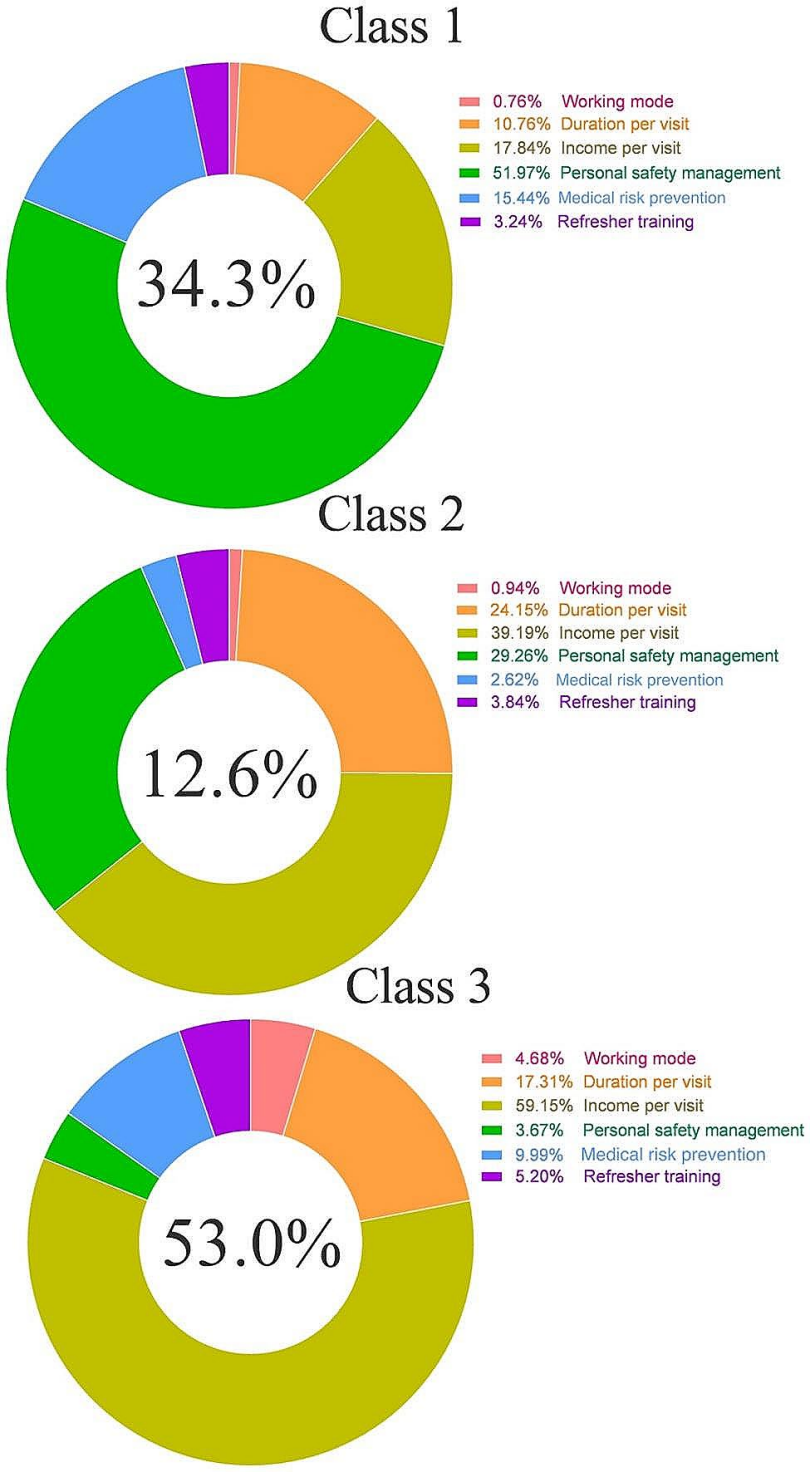
The proportion of each attribute for each class

According to the Bayesian standard minimum, there were three classes in this study, with Fig. 2 displaying the percentage of each class.

**Fig. 3:**
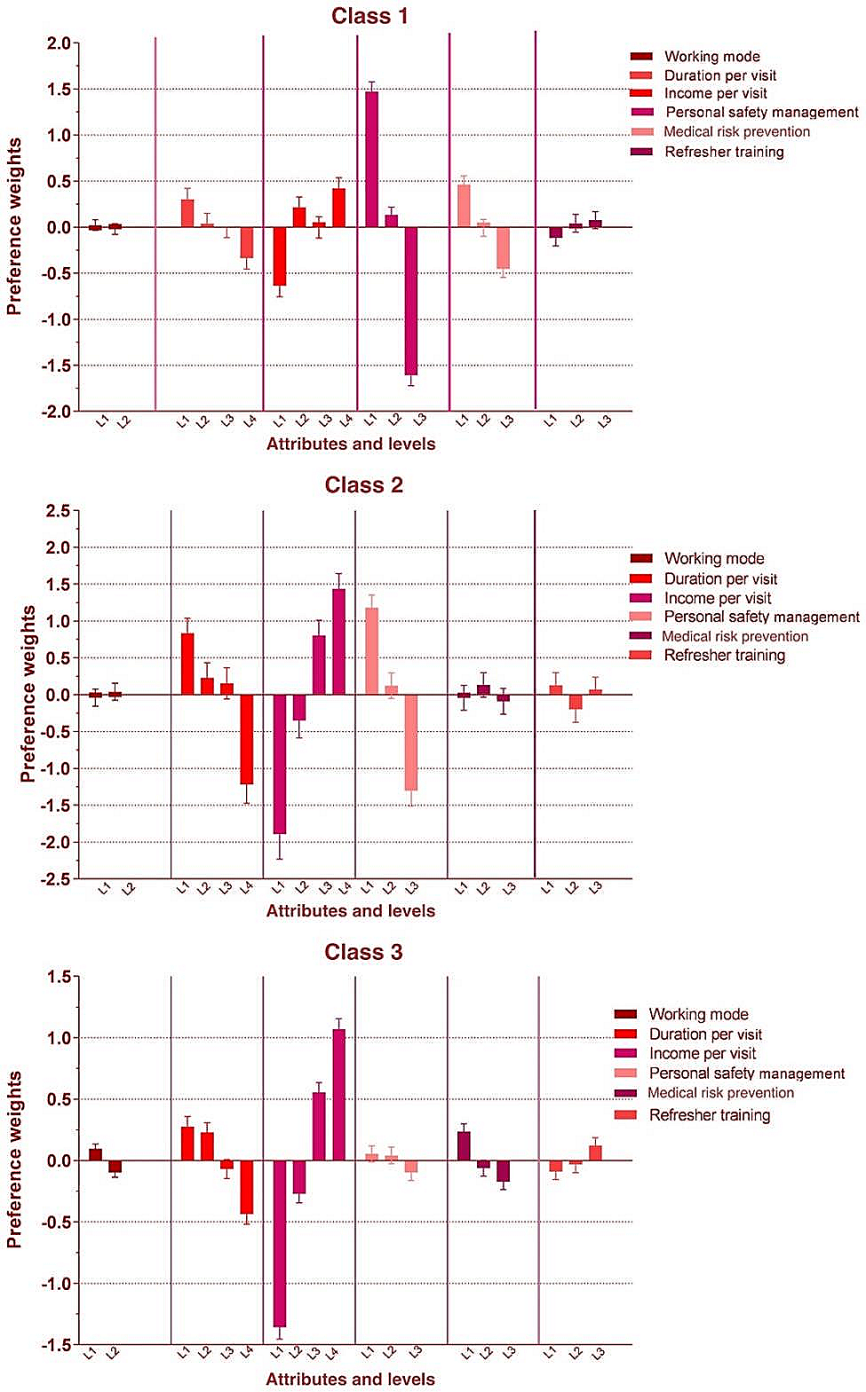
Latent percentage weights for three classes

In the first class (Fig. 3), personal safety management accounted for 51.97% of top concerns. Sharing nurses, with a preference weight of -1.60 to 1.47, wanted the highest level of personal safety while working. In the second class (Fig. 3), the most important attribute was income per visit with 39.19%, statistically significant except for the level of 250, with a preference weight of -1.89 to 1.44. The second most important category was personal safety management with 29.26%, and a preference weight of -1.30 to 1.18, i.e., that sharing nurses want to be paid higher for their work, and also get a higher level of protection for personal safety. With a preference weighting of -1.36 to 1.07, statistically significant at all levels. The third class (Fig. 3) was comparable to the second class as it similarly placed a greater emphasis on income per visit at 59.15%. However, they have less concern for personal safety management, with a percentage of 3.67 and a preference weight of -0.10 to 0.05.

### Willingness to pay

We summarized participants’ willingness to pay and the three classes into which they were classified (Table 5). The results showed that, in general, sharing nurses were willing to give up an average of 46.36 CNY in exchange for a higher level of personal safety management. Also they were also willing to earn less (WTP=-17.44) to make the medical risk preventions go from low level to higher level. The first class of sharing nurses, who sought a higher level of personal safety, accepted a higher level despite a correspondingly lower income (WTP=-209.80). Moreover, there was no resistance to a higher level of medical risk prevention with lower remuneration (WTP=-62.32). The value that the second-class places on full-time work is higher (WTP = 1.73), which was not preferred by the other two classes. The third and first class had similar characteristics but with lower willingness to reduce their benefits with higher levels of personal safety (WTP=-4.47), and medical risk prevention (WTP=-12.16).

**Table 5:** **Respondents’ WTP** ^a,b^

Attributes	Overall WTP (N = 220), ¥ (US $)	WTP in class 1 (n = 75), ¥ (US $)	WTP in class 2 (n = 28), ¥ (US $)	WTP in class 3 (n = 117), ¥ (US $)
Working mode	-0.10(-4.00)	-0.05(-3.08)	0.08(1.73)	-0.19(-5.69)
Duration per visit	-0.23(-9.19)	-0.21(-14.47)	-0.68(-14.79)	-0.24(-7.02)
Income per visit(¥)a	Reference	Reference	Reference	Reference
Personal safety management	-1.16(-46.36)	-3.08(-209.80)	-2.49(-53.76)	-0.15(-4.47)
Medical risk prevention	-0.44(-17.44)	-0.91(-62.32)	-0.05(-1.03)	-0.41(-12.16)
Refresher training	0.11(4.35)	0.19(13.06)	-0.06(-1.26)	0.21(6.33)

^a^ WTP: willingness to pay

^b^ A negative dollar amount shows the cost the respondent is willing to pay to move up a level

## Discussion

### Principal findings

In this study, we aimed to use a discrete choice experiment to explore the relative preferences of nurses regarding attributes of the IPNS job. We found that participants had significant preferences for income and personal safety management, which means higher income is an important motivation for nurses to participate in the IPNS program. In addition to this, nurses preferred to offer home nursing services with good personal safety measures and medical risk prevention. Among the non-economical factors, visiting duration and working modes were also among the nurses’ considerations. Overall, sharing nurses preferred to be paid for a short visiting duration with a part-time mode and received as much training as possible with reliable security and medical risk prevention, both economical and non-economical incentives are factors that nurses consider when joining the IPNS program.

It is not surprising that income is most important for sharing nurses. Participants with full-time jobs in health institutions who offer home health care services in their spare time deem it essential to be paid what they deserve, considering their time and effort [30]. As sharing nurses traveling to clients’ homes with inconsistent workloads, they must sometimes offer extra work without pay or pay for transport costs and work-related expenses with their own money. Previous research found that salary is an important factor in the retention of home health care nurses and that a stable income attracts nurses [31, 32]. This explains why participants prefer part-time to full-time nursing jobs, and the stability of their original job in a healthcare facility and steady income are preferred [33, 34].

Besides income, nurses are concerned about personal safety and a unique home-based work environment [35, 36]. Personal safety precautions included pre-visits and in-visit measures, such as client information verification, home environment assessment, alarm equipment, real-time position tracking, and procedure recording. These measures were insufficient to alleviate nurses’ concerns about their safety and must be developed for improved effectiveness. Tourangeau et al. found that home safety is a concern about which nurses care and is consistent with our findings [32].

In contrast to previous studies, participants were not sensitive to refresher training compared with other attributes. Cooper et al. [37] found that adequate training ensured that nurses would carry out home care services. Our findings found there was no significant preference for more training, which may be related to the policy in Guangdong regarding how sharing care nurses must attend a series of well-designed courses and pass tests to acquire the IPNS qualification. As such, refresher training had no significant effect on nurses’ preferences.

In this study, LCA was used to explore the heterogeneity of individual preferences of different nurses and found that nurses could be categorized into three different classes based on their preferences. Nurses in the first class had the highest demand for personal safety, while nurses in the second class were concerned about income as well as personal safety, and the third class also valued income, but not as concerned about personal safety in comparison to the other two classes. Personal safety was among the most frequently cited factors in previous nursing studies [32]. It is reasonable that most sharing nurses valued personal safety, as there were potential hazards associated with working in a client’s home, e.g., working alone, unsafe environments, nonfamiliarity with clients, and pets.

### Research implications

The Internet Plus Nursing Service program enabled the rapid spread of home care services in China by combining mobile device technology and care resources. As the demand for home health care increased, the shortage of sharing nurses has become a concern. The results suggested that income is one of the most important factors for sharing nurses while making job decisions, thus it is necessary for medical organizations to ensure nurses receive an income worthy of the services they’ve offered. Attention should also be paid to the safety issues and risk faced by nurses, and relevant policies should be developed to address nurses’ concerns. Moreover, a manageable workload and systematic trainings may be measures that could facilitate nurses’ participation in IPNS program. Our findings serve as a reminder that decision-makers should take both economic and non-economic incentives into account to improve the working environment for sharing nurses.

### Strengths and limitations

This is one of the first studies to analyze job preferences for IPNS nurses, using a DCE approach, offering insight into factors that influence nurses’ work decisions. Currently, one study [17] have assessed nurses’ perceptions of the IPNS program, concluding that nurses are optimistic about this program, which still needs refinements. In this study, we used a qualitative approach to explore processes that lead to nursing job choices and a quantitative approach to analyze the weighting of preferences, informing policymakers that the development of both economic and non-economical strategies is important.

There are some limitaitons in our study, the first one is about sample representativeness; we mainly surveyed medical institutions in Guangzhou, but nurses’ preferences varied in different regions. Larger sample size and more diverse allocation method would obtain reliable results in the LCA. Secondly, several nurses refused to take the survey or were not able to complete the questionnaire, and it is possibly due to time constraints or busy schedules. Although the response rate was more than 95% (95.24%) in this study, possible selection bias caused by the fact that some individuals refused to respond should still be considered. Thirdly, indirect effects are often described by calculating the interaction between different attributes, which was not carried out in this study due to insufficient sample size, so it may have an impact on the interpretation of the results. Furthermore, although we used a qualitative research approach to ensure that we included the key attributes, there are still other factors that may influence shargin nurses’ job choices, and future research could be conducted on this.

## Conclusions

Understanding the job preferences of sharing nurses is essential to address the manpower shortage in the IPNS program. The results of this study suggest that sharing nurses tended to choose jobs with higher incomes, and other attributes including superior safety management, higher levels of medical risk prevention, and shorter visiting duration also have an impact on nurses’ job choice. The apparent preference of sharing nurses for income suggests the need to provide nurses with a reasonable salary to make them feel rewarded for their efforts. In addition, it should also take into account the provision of higher levels of security and medical risk prevention, given nurses’ concerns about safety and risk. Ensuring sufficient infrastructural and organizational support, such as refresher training and flexible working mode, also help improve nurse recruitment and prevent turnover. While developing strategies to engaging nurses in the IPNS program and home health care, decision-makers should not only consider economic factors but also non-economic factors to gain sharing nurses’ recognition for the work.

## Data Availability

The datasets collected and/or analyzed during the current study are available from the corresponding author upon reasonable request.

## Abbreviations

IPNS: the Internet Plus Nursing Service
DCE: Discrete choice experiment
LCA: Latent class analysis
BIC: Bayesian Information Criteria

## References

[1] Fang EF, Xie C, Schenkel JA, Wu C, Long Q, Cui H (2020). A research agenda for ageing in China in the 21st century (2nd edition): focusing on basic and translational research, long-term care, policy and social networks. Ageing Res Rev.

[2] Wang Y, Wang L, Qu W (2017). New national data show alarming increase in obesity and noncommunicable chronic Diseases in China. Eur J Clin Nutr.

[3] WHO. Year of the nurse and the midwife 2020 2020 [Available from: https://www.who.int/campaigns/annual-theme/year-of-the-nurse-and-the-midwife-2020.

[4] Du Y, Shi X, Zhang W, Wang Y, Jia X, Cheng Z (2018). Exploration on the trend of temporal and spatial distribution of population aging in China. Chin J Health Stat.

[5] Yang G, Wang Y, Zeng Y, Gao GF, Liang X, Zhou M (2013). Rapid health transition in China, 1990–2010: findings from the global burden of Disease Study 2010. Lancet.

[6] Commission NH. Centers for Disease Control and Prevention: China Health Standard Management; 2017 2017 [Available from: https://www.cdc.gov/nchs/fastats/home-health-care.htm.

[7] Care ANATFtDSoNPfHH, Committee CoCHNE. Standards of home health nursing practice. American Nurses’ Association; 1986.

[8] Dieckmann JL. Home Health Care: A Historical. Handbook of home health care administration. 2015:9.

[9] Bureau of Labor Statistics. Career outlook: projections of industry employment: 2014–2024 2025 [Available from: http://www.bls.gov/careeroutlook/2015/article/projections-industry.htm.

[10] Genet N, Boerma W, Kroneman M, Hutchinson A, Saltman RB, Organization WH. Home care across Europe: current structure and future challenges. World Health Organization. Regional Office for Europe; 2012.

[11] Moriyama M (2008). Family nursing practice and education: what is happening in Japan?. J Fam Nurs.

[12] Morioka N, Okubo S, Yumoto Y, Ogata Y (2019). Training opportunities and the increase in the number of nurses in home-visit nursing agencies in Japan: a panel data analysis. BMC Health Serv Res.

[13] Ma W, Meng X, Wei Y, Li J (2019). Roles and activities of community nurses in China: a descriptive study. J Nurs Adm Manag.

[14] Zhu H, Lu J, Zhang Y, Cui B (2019). Responses to population ageing in the new era: a national condition report from China. China Popul Dev Stud.

[15] China Healthcare Commission. China pilots an internet plus nursing program 2019 2019 [Available from: http://english.www.gov.cn/state_council/ministries/2019/02/13/content_281476520056360.htm.

[16] China GOvernment Network. Chinese ‘nurse sharing’ app to offer at-home care 2018: 2018; 2018 [Available from: http://www.ecns.cn/news/society/2018-05-30/detail-ifyuurnp0995009.shtml.

[17] Huang R, Xu M, Li X, Wang Y, Wang B, Cui N (2020). Internet-based sharing nurse program and nurses’ perceptions in China: cross-sectional survey. J Med Internet Res.

[18] Lancsar E, Louviere J (2008). Conducting discrete choice experiments to inform healthcare decision making: a user’s guide. PharmacoEconomics.

[19] Kjaer, T. A review of the discrete choice experiment-with emphasis on its application in health care. Denmark: Syddansk Universitet, 2005.

[20] Ryan M (1999). Using conjoint analysis to take account of patient preferences and go beyond health outcomes: an application to in vitro fertilisation. Soc Sci Med.

[21] Reed Johnson F, Lancsar E, Marshall D, Kilambi V, Mühlbacher A, Regier DA (2013). Constructing experimental designs for discrete-choice experiments: report of the ISPOR Conjoint Analysis Experimental Design Good Research practices Task Force. Value Health.

[22] Huber J, Zwerina K (1996). The importance of utility balance in efficient choice designs. J Mark Res.

[23] Guimarães C, Marra CA, Gill S, Simpson S, Meneilly G, Queiroz RH et al. A discrete choice experiment evaluation of patients’ preferences for different risk, benefit, and delivery attributes of insulin therapy for diabetes management. Patient preference and adherence. 2010:433 – 40. 10.2147/PPA.S14217 PMC3034358 21301591

[24] Porteous T, Ryan M, Bond CM, Hannaford P (2006). Preferences for self-care or professional advice for minor Illness: a discrete choice experiment. Br J Gen Pract.

[25] Koo W, Kim H (2020). Bayesian nonparametric latent class model for longitudinal data. Stat Methods Med Res.

[26] Goossens LM, Utens CM, Smeenk FW, Donkers B, van Schayck OC, Rutten-van Mölken MP (2014). Should I stay or should I go home? A latent class analysis of a discrete choice experiment on hospital-at-home. Value in Health.

[27] Greene WH, Hensher DA (2003). A latent class model for discrete choice analysis: contrasts with mixed logit. Transp Res Part B: Methodological.

[28] Clark MD, Determann D, Petrou S, Moro D, de Bekker-Grob EW (2014). Discrete choice experiments in health economics: a review of the literature. PharmacoEconomics.

[29] Bridges JF, Hauber AB, Marshall D, Lloyd A, Prosser LA, Regier DA (2011). Conjoint analysis applications in health–a checklist: a report of the ISPOR Good Research Practices for Conjoint Analysis Task Force. Value Health.

[30] Mabry L, Parker KN, Thompson SV, Bettencourt KM, Haque A, Luther Rhoten K (2018). Protecting workers in the home care industry: workers’ experienced job demands, resource gaps, and benefits following a socially supportive intervention. Home Health Care Serv Q.

[31] Armstrong-Stassen M, Cameron SJ (2005). Concerns, satisfaction, and retention of Canadian community health nurses. J Commun Health Nurs.

[32] Tourangeau A, Patterson E, Rowe A, Saari M, Thomson H, MacDonald G (2014). Factors influencing home care nurse intention to remain employed. J Nurs Adm Manag.

[33] Ellenbecker CH, Porell FW, Samia L, Byleckie JJ, Milburn M (2008). Predictors of home healthcare nurse retention. J Nurs Scholarsh.

[34] Tourangeau AE, Cummings G, Cranley LA, Ferron EM, Harvey S (2010). Determinants of hospital nurse intention to remain employed: broadening our understanding. J Adv Nurs.

[35] Anthony A, Milone-Nuzzo P (2005). Factors attracting and keeping nurses in home care. Home Healthc Nurse.

[36] Palumbo MV, McIntosh B, Rambur B, Naud S (2009). Retaining an aging nurse workforce: perceptions of human resource practices. Nurs Econ.

[37] Cooper C, Cenko B, Dow B, Rapaport P (2017). A systematic review evaluating the impact of paid home carer training, supervision, and other interventions on the health and well-being of older home care clients. Int Psychogeriatr.

